# Membrane binding and bending in Ebola VP40 assembly and egress

**DOI:** 10.3389/fmicb.2014.00300

**Published:** 2014-06-18

**Authors:** Robert V. Stahelin

**Affiliations:** ^1^Department of Biochemistry and Molecular Biology, Indiana University School of Medicine-South BendSouth Bend, IN, USA; ^2^Department of Chemistry and Biochemistry, Eck Institute for Global Health, University of Notre DameNotre Dame, IN, USA

**Keywords:** Ebola, filovirus, membrane binding, plasma membrane, phosphatidylserine, phosphoinositides, VP40

## Abstract

Lipid-enveloped viruses contain a lipid bilayer coat that protects their genome and helps to facilitate entry into the host cell. Filoviruses are lipid-enveloped viruses that have up to 90% clinical fatality and include Marbug (MARV) and Ebola (EBOV). These pleomorphic filamentous viruses enter the host cell through their membrane-embedded glycoprotein and then replicate using just seven genes encoded in their negative-sense RNA genome. EBOV budding occurs from the inner leaflet of the plasma membrane (PM) and is driven by the matrix protein VP40, which is the most abundantly expressed protein of the virus. VP40 expressed in mammalian cells alone can trigger budding of filamentous virus-like particles (VLPs) that are nearly indistinguishable from authentic EBOV. VP40, such as matrix proteins from other viruses, has been shown to bind anionic lipid membranes. However, how VP40 selectively interacts with the inner leaflet of the PM and assembles into a filamentous lipid enveloped particle is mostly unknown. This article describes what is known regarding VP40 membrane interactions and what answers will fill the gaps.

## INTRODUCTION

The discovery of filoviruses, Marburg virus (MARV) and Ebola virus (EBOV) in 1967 and 1976, respectively, spread fear of new pandemics that could spread globally and kill millions of people (Dowdle, 1976; Johnson et al., 1977). While outbreaks have been smaller for filoviruses, the fatality can be as high as 90% and there is some concern that a more significant pandemic could be looming. EBOV and MARV are also classified as category A pathogens by the NIH, a designation indicating they pose the highest risk to public safety and national security. In addition, filoviruses may be found outside of sub-Saharan Africa (Marsh et al., 2011; Negredo et al., 2011; Yuan et al., 2012), further underscoring the need for new treatment options. To date, therapeutics or vaccines have yet to be approved by the FDA for EBOV, but great strides have been made toward this goal (Geisbert et al., 2010; Blaney et al., 2013; Johansen et al., 2013; Pettitt et al., 2013; Warren et al., 2014).

Filoviruses are lipid-enveloped, filamentous in shape, and harbor a negative-sense RNA genome. The genome encodes seven proteins including nucleoprotein (NP), VP24, VP30, VP35, and L protein, which constitute the nucleocapsid (NC; Olejnik et al., 2011; Booth et al., 2013). The transmembrane glycoprotein (GP) is rooted in the lipid envelope of the virus and is responsible for entry of virions into the host cell (Lee et al., 2008; Cote et al., 2011; Olejnik et al., 2011). VP40 (viral protein 40 kDa) is the EBOV matrix protein, which regulates viral budding and NC recruitment as well as virus structure and stability. Sole expression of VP40 in mammalian cells is enough to assemble and form virus-like particles (VLPs) that are similar in size, shape, and nearly indistinguishable from the authentic virus (Jasenosky et al., 2001; Timmins et al., 2001, 2003; Noda et al., 2002, 2006; Licata et al., 2004). VP40 consists of 326 amino acids and has been shown to be a peripheral protein (Jasenosky et al., 2001), which localizes to the inner leaflet of the plasma membrane (PM) of human cells. Here, VP40 oligomers guide formation of new viral particles (Timmins et al., 2003; Adu-Gyamfi et al., 2012a, 2013; Bornholdt et al., 2013). Although the molecular basis of VP40 membrane binding is not well understood, mutations of VP40 that abrogate PM localization (McCarthy et al., 2007; Adu-Gyamfi et al., 2013; Bornholdt et al., 2013) or PM insertion (Adu-Gyamfi et al., 2013; Soni et al., 2013) inhibit viral budding.

## PLASMA MEMBRANE LIPIDS

**FIGURE 1:**
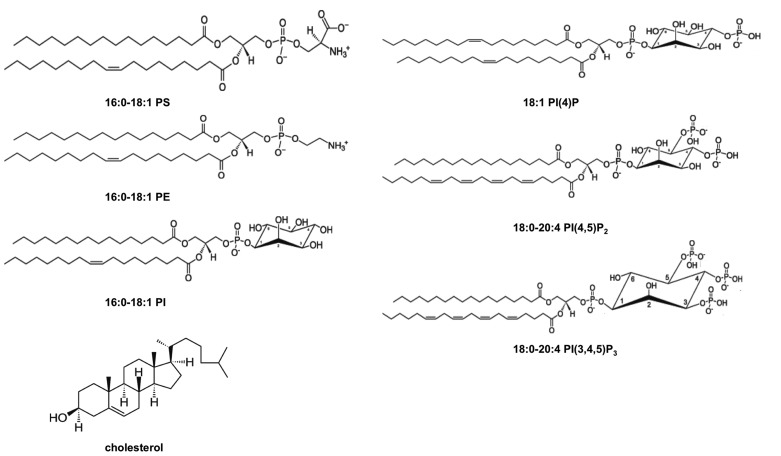
**Chemical structures of lipids found in the PM cytoplasmic leaflet**. The inner leaflet of the PM has a relatively high concentration of PE and PS and it is known to be enriched in cholesterol and PI. Additionally, PIPs including PI(4)P, PI(4,5)P_2_, and PI(3,4,5)P_3_ are found in low abundance in the PM inner leaflet but have crucial roles in regulating peripheral protein localization and function at the membrane interface. Structures are shown at physiological pH.

The PM is an asymmetric bilayer that is approximately 30 Å thick and contains ~15% of its mass as transmembrane protein. A hallmark of PM asymmetry is an outer leaflet composed mainly of phosphatidylcholine (PC), sphingomyelin (SM), and glycosphingolipids and an inner leaflet enriched with phosphatidylethanolamine (PE), phosphatidylserine (PS), phosphatidylinositol (PI), and phosphoinositides (PIPs; Schick et al., 1976; Higgins and Evans, 1978; Venien and Le Grimellec, 1988; van Meer et al., 2008; see **Figure 1**). Additionally, the PM is enriched in cholesterol and by some accounts may contain as much as 50% of cholesterol (van Meer et al., 2008) although the distribution of cholesterol among the outer and inner leaflets is not as well understood. The PM inner leaflet contains ~20–30 mol% anionic lipid (McLaughlin et al., 2002; McLaughlin and Murray, 2005; Vance and Steenbergen, 2005; Vance and Tasseva, 2013), which can attract peripheral proteins with cationic patches and selective binding domains. The enrichment of PIPs, including PI(4,5)P_2_ (McLaughlin et al., 2002; McLaughlin and Murray, 2005; Balla, 2013), PI(4)P (Hammond et al., 2012), and PI(3,4,5)P_3_ (Heo et al., 2006) make up a small fraction of phospholipids on the inner leaflet but play an important role in recruiting high-affinity targets and mediating electrostatic interactions.

PS is the most abundant anionic lipid in the cytosolic leaflet at ~15–20% (Vance and Steenbergen, 2005; Yeung et al., 2008; Vance and Tasseva, 2013) and contributes to the recruitment of polycationic proteins as well as proteins containing specific PS binding domains (Cho and Stahelin, 2005, 2006). PS has also been shown to interact with several viral matrix proteins (Zakowski et al., 1981; Dick et al., 2012) as well as VP40 (Ruigrok et al., 2000; Scianimanico et al., 2000; Adu-Gyamfi et al., 2013; Soni et al., 2013). The coexistence of PS and PIPs in the same membrane provides a mode of regulation for peripheral proteins that bind weakly to just one anionic lipid. Coincidence detection, which refers to recognition of two or more distinct membrane lipids (Balla, 2005; Lemmon, 2008; Moravcevic et al., 2012; Scott et al., 2012; Stahelin et al., 2014) has recently become a popular model of peripheral protein recruitment to biological membranes including that of HIV-1 Gag (Vlach and Saad, 2013). Additive and synergistic effects of either the anionic charge at the PM, the presence of two distinct binding sites (e.g., PI(4,5)P_2_ and PS), or binding to a lipid in a region of positive curvature can mediate the selective PM localization of some peripheral proteins.

Membrane rafts are a common and controversial model of membrane-related research. Rafts are enriched in cholesterol and ordered membrane lipids with high-melting (*T*_m_) temperatures (Komura and Andelman, 2013; Sonnino and Prinetti, 2013). Lipids are dynamic in bilayers and can move and rotate in the plane of the membrane, which can change with variation in lipid composition and temperature. Membrane phase behavior describes these lipid properties and is defined as having the state of liquid-ordered (L_o_), liquid-disordered (L_d_), or solid-gel (L_β_). The rafts in membranes refer to the coexistence of the L_o_ and L_d_ phases. L_o_ phases are enriched with cholesterol and lipids with saturated acyl chains or a mixture of saturated and unsaturated acyl chains (Sonnino and Prinetti, 2013). L_d_ phases, on the other hand, harbor unsaturated acyl chains and lower concentrations of cholesterol. The unsaturated acyl chains are kinked and more spread out, leading to a more loosely packed bilayer. The loose packing is not as favorable to shield cholesterol when compared to that of an ordered and saturated membrane.

Several viruses including HIV (Ono and Freed, 2001; Brugger et al., 2006; Waheed and Freed, 2009) and EBOV (Panchal et al., 2003) have been suggested to bud from raft regions of the PM. This is because viruses have been shown to be enriched with cholesterol and sphingomyelin, be dependent upon membrane cholesterol levels for budding (Ono et al., 2007), and associate with the detergent-resistant fraction (Panchal et al., 2003) of cellular membranes commonly associated with rafts. The PM outer leaflet can harbor coexistence of L_o_ and L_d_ phases but as recently and elegantly discussed in the context of HIV-1 Gag (Dick et al., 2012), it is important to note that the coexistence of L_d_ + L_o_ phases has not been detected in bilayer models that more closely resemble the PM inner leaflet (Wang and Silvius, 2001). Admittedly, *in vitro* models are limited to more chemically simple lipid mixtures than biological membranes but there are still many questions in this field including whether viral proteins themselves can induce raft-like properties in the membrane.

## VP40 INTERACTIONS WITH MEMBRANES

### THE VP40 C-TERMINAL DOMAIN ASSOCIATES WITH PS-CONTAINING MEMBRANES

The first VP40 X-ray structure (Dessen et al., 2000) revealed a matrix protein with an N-terminal domain that is important for oligomerization (Timmins et al., 2003; Bornholdt et al., 2013) and a C-terminal domain now shown to be essential to membrane binding (Ruigrok et al., 2000; Scianimanico et al., 2000; Jasenosky et al., 2001; Adu-Gyamfi et al., 2013; Bornholdt et al., 2013; Soni et al., 2013). Ruigrok et al. (2000) first demonstrated that VP40 associated with lipid vesicles composed of 25% PC, 50% PS, and 25% cholesterol using a VP40 construct containing residues 31-326 (see also **Table 1**). They also demonstrated that the VP40 C-terminal domain was an important determinant of membrane binding as a truncated construct (residues 31-212) has greatly reduced binding to the same PS-containing vesicles. In these studies, 1 M NaCl was also able to reduce association with the vesicles containing 50% PS. Additionally, vesicles containing only 5% PS did not support significant VP40 binding. Taken together, C-terminal domain electrostatic interacts seem to be a key determinant of anionic membrane association.

**Table 1:** Summary of VP40 mutations and truncations that have been assessed in the context of membrane binding or plasma membrane association.

VP40 mutation or truncation	*In vitro* membrane-binding effects	Cellular ‘effects	Reference
VP40 31-326	Associated with liposomes containing 50% PS and only weakly with liposomes containing 5% PS; 1 M NaCl significantly reduced association with liposomes containing 50% PS	Not measured	Ruigrok et al. (2000)
VP40 31-326	Liposome association induces hexamerization	Not measured	Scianimanico et al. (2000)
VP40 31-319	*In vitro* liposome binding was enhanced and induced hexamerization	Not measured	Scianimanico et al. (2000)
VP40 1-326	Not measured	A peripheral protein in the aqueous phase of a TX-114 extraction; 1 M NaCl did not perturb cellular membrane interactions indicating hydrophobic interactions are important; oligomers associated with membrane rafts	Jasenosky et al. (2001), Panchal et al. (2003)
VP40 1-276	Not measured	Greatly reduced association with cellular membranes and no VLPs; membrane association was sensitive to 1 M NaCl	Jasenosky et al. (2001)
VP40 1-318	Not measured	Less PM association and VLP formation	Panchal et al. (2003)
VP40 1-308	Not measured	Globular oligomeric cytosolic structures observed; no VLP formation	Panchal et al. (2003)
AAXY (P10A/P11A)	Not measured	Associates with membrane the same as WT but cannot form VLPs	Jasenosky et al. (2001)
W95A	Not measured	Reduces oligomerization and budding	Hoenen et al. (2010)
T112R	Abolishes dimerization, forms monomers and rings	Undetectable PM localization and budding	Bornholdt et al. (2013)
L117R	Abolishes dimerization, forms monomers and rings	Undetectable PM localization and budding	Bornholdt et al. (2013)
R134A	Abolishes RNA binding	Similar budding and PM localization; abolishes RNA binding	Bornholdt et al. (2013)
212-KLR-214 mutations	Not measured	Altered cellular localization and oligomerization; greatly reduced budding	McCarthy et al. (2007)
L213A	Reduced penetration into plasma membrane mimetic	Reduced PM localization and oligomerization	McCarthy et al. (2007), Adu-Gyamfi et al. (2013)
Δ221-229	Not measured	Abolished budding and PM localization	Bornholdt et al. (2013)
K224E/K225E	Not measured	Abolished budding and PM localization	Bornholdt et al. (2013)
K224M/K225M	Not measured	Abolished budding and PM localization	Bornholdt et al. (2013)
K224R/K225R	Not measured	Restored budding and PM localization	Bornholdt et al. (2013)
M241R	Twisted hexamer is formed	PM localization and membrane ruffling but no VLP formation	Bornholdt et al. (2013)
K274A/275A	Not measured	Reduced budding but retains PM localization	Bornholdt et al. (2013)
K274R/275R	Not measured	Restores budding	Bornholdt et al. (2013)
P283L/P286L	Not measured	Cytosolic oligomers in the detergent soluble fraction; no VLP formation	Panchal et al. (2003)
I293A	Reduced penetration into PM mimetic	Greatly reduced PM localization, oligomerization and budding	Adu-Gyamfi et al. (2013)
L295A	Reduced penetration into PM mimetic	Greatly reduced PM localization, oligomerization and budding	Adu-Gyamfi et al. (2013)
V298A	Reduced penetration into PM mimetic	Greatly reduced PM localization, oligomerization, and budding	Adu-Gyamfi et al. (2013)
A299W	Used to assess VP40 depth of membrane penetration (8.1 Å) and binds membranes similar to WT	Localizes and buds similar to WT	Soni et al. (2013)
I293A/A299W	Significantly reduced membrane penetration	Reduced PM localization and abolished budding	Soni et al. (2013)
L295A/A299W	Significantly reduced membrane penetration	Reduced PM localization and abolished budding	Soni et al. (2013)
I307R	Induces ring formation	No budding or PM localization. Globular perinuclear structures observed.	Bornholdt et al. (2013)

Lipid vesicles containing PS have also been shown to induce a conformational change in VP40 that results in hexamerization (Scianimanico et al., 2000). This study used the same lipid composition noted above (25% PC, 50% PS, and 25% cholesterol) and two VP40 constructs (31-319 and 31-326). Cross-linking demonstrated dimer, trimer, tetramer, and hexamers formed for the VP40 31-319 construct. Cross-linking with PS liposomes exhibited no detectable monomer but rather extensive oligomerization including the large hexamer as the predominant band. It is also important to note that they found 1% β-octyl-glucoside was needed to avoid extensive VP40 aggregation in these assays. Hexamers were also found exclusively localized in filopodia-like projections emanating from the PM of human cells (Adu-Gyamfi et al., 2012a). In these studies, total internal reflection fluorescence (TIRF) microscopy was used to selectively excite fluorophores on or near the PM interface. VP40 assembly and oligomerization into hexamers and larger oligomers was clearly dependent on association with the PM interface.

### VP40 C-TERMINAL DOMAIN HYDROPHOBIC INTERACTIONS ARE IMPORTANT FOR VLP FORMATION

In 2001, it was shown that VP40 alone formed VLPs from cells (Jasenosky et al., 2001). These studies investigated VP40 and respective truncation constructs’ ability to associate with cellular membranes. Triton X-114 detergent extraction was done in order to separate the aqueous and detergent phase to investigate the mode of VP40 PM association. Integral membrane proteins and lipidated proteins are found in the detergent phase, while peripheral proteins reside in the aqueous phase of these extractions. VP40 was found to be a peripheral protein as it was almost exclusively extracted in the aqueous phase. Notably, these authors propose the important role of hydrophobic interactions of VP40 in membrane binding as they find 1 M NaCl did not release VP40 from the membrane bilayer. A hydropathy plot demonstrated that the majority of VP40 hydrophobic residues (Jasenosky et al., 2001) were in the last 50 residues of the C-terminal domain, which was further supported with a VP40 1-276 truncation. The low-to-moderate PM association of this truncation (VP40 1-276) was perturbed by an increase in the salt concentration. This is in contrast to the study discussed above that demonstrated VP40 association with membranes was driven by electrostatic interactions (Ruigrok et al., 2000). Note, however, that those studies were done with PS-containing liposomes and VP40 was not completely displaced from the vesicles. Additionally, the PM bilayer is much more complex than PC/PS/cholesterol liposomes and hydrophobic interactions may play a more prominent role in locking VP40 into the PM interface, where VP40 stays until the virions infect a new round of cells.

### VP40 OLIGOMERS ASSOCIATE WITH PLASMA MEMBRANE RAFTS

Membrane rafts have also been implicated in VP40 assembly and oligomerization at the PM (Panchal et al., 2003). VP40 oligomers were found exclusively associated in the membrane fraction, while the soluble cytosolic fraction contained mostly monomeric VP40. The raft fractions, which are also referred to as detergent-resistant membranes (DRM), showed enrichment of VP40 oligomers. Several truncations of VP40 were prepared including a 9-residue C-terminal deletion (1-317) and an 18-residue C-terminal deletion (1-308). The authors proposed that the C-terminal 9 amino acids likely contribute to the membrane binding as this truncation had less PM localization than wild type (WT). The 18-residue C-terminal deletion had undetectable PM localization and formed globules in the cytoplasm, which may be a sign of octameric ring formation. Octamer formation has been shown to occur in some mutations of the C-terminal domain, resulting in perinuclear globules (Bornholdt et al., 2013). This 18-residue truncation additionally was shown to be oligomeric, associated with the detergent soluble fraction, and was unable to make VLPs (Panchal et al., 2003).

The authors identified proline 283 and proline 286 akin to an SH3 domain-binding site so they were mutated to leucine (Panchal et al., 2003). This resulted in oligomers in the cell lysate and the detergent soluble fraction but exclusion from DRM. The proline double mutant formed large aggregates in the cellular cytoplasm as well. Additionally, no VLPs were generated for P283L/P286L. Panchal et al. proposed that association with DRM is required for release of VP40 VLPs and that residues 309–317 as well as Pro^283^ and Pro^286^ are crucial for VP40 association with the PM microdomains. It still is unclear how rafts, which are relatively small in size compared to the EBOV virions, regulate budding of filamentous particles that are a micron or more in length. Moreover, the PM inner leaflet has not been shown to harbor a raft-like composition further confounding how VP40 may assemble and bud. One interesting and somewhat related possibility is the location of PS on the PM inner leaflet. Recently, it was shown using the robust PS sensor lactadherin C2 that PS is clustered and associated with caveolae in the PM inner leaflet (Fairn et al., 2011), which may explain VP40 oligomers being localized to DRM. A careful examination of the EBOV lipidome would help resolve some of these questions.

### VP40 CAN PENETRATE INTO THE PLASMA MEMBRANE HYDROCARBON CORE

To investigate if hydrophobic interactions are important for VP40 association with membranes *in vitro*, a lipid monolayer assay was employed (Adu-Gyamfi et al., 2013). Lipid monolayers are a useful platform for assessing the ability of peripheral proteins to penetrate into membranes of a lipid composition the experimentalist chooses (Cho et al., 2001). Biological membranes and lipid vesicles have surface pressures in the 30–35 mN/m range (Demel et al., 1975; Marsh, 1996), so proteins that are thought to penetrate under physiological conditions usually have a critical pressure (π_c_) above 30 mN/m. The ability of VP40 to penetrate into monolayers that served as a mimetic of the cytoplasmic face of the PM (PC:PE:PS:PI:cholesterol (12:35:22:9:22)) or the nuclear envelope (PC:PE:PS:PE:cholesterol (61:21:4:7:7); Stahelin et al., 2003b) was assessed. In brief, VP40 was able to penetrate significantly into the PM mimetic (π_c_ ~ 34 mN/m) compared to that of the nuclear membrane mimetic (π_c_ ~ 25 mN/m; Adu-Gyamfi et al., 2013).

To investigate the molecular basis of VP40-mediated penetration, the authors used the rationale that the C-terminal domain undergoes hydrophobic interactions with the membrane bilayer within the last 50 amino acids (Jasenosky et al., 2001). Examining the structure of the VP40 C-terminal domain revealed a loop region rich in hydrophobic residues (Ile^293^, Leu^295^, and Val^298^) conserved among the EBOV strains. A control mutation of Leu^303^ that was previously shown to interact with Sec24C (Yamayoshi et al., 2008) was also prepared. It was also noted that Leu^213^, which was previously shown to be important for VP40 PM localization and oligomerization (McCarthy et al., 2007), was on the same interface as Ile^293^, Leu^295^, and Val^298^. Thus, L213A was also prepared. L213A, I293A, L295A, and V298A all exhibited greatly reduced penetration into the PM mimetic indicating these residues are important for docking into lipid membranes. L303A, however, did not influence the ability of VP40 to penetrate into the PM mimetic. The effects of these hydrophobic mutations on VP40 PM localization, oligomerization in cells, and the ability to form VLPs were examined. In consonance with *in vitro* penetration data L213A, I293A, L295A, and V298A all had significantly reduced PM localization. This indicated that membrane penetration by this hydrophobic interface is important for the localization and latching on of VP40 to the PM bilayer. Single-molecule imaging using TIRF demonstrated that membrane penetration is an important step in VP40 oligomerization as these mutations abolished detectable hexamerization of VP40. Finally, these mutations also had greatly diminished VLP formation signifying the importance of this region in filamentous particle formation. The control mutation, L303A, behaved similar to WT VP40 in all cellular experiments. Importantly, hydrophobic mutations did not exhibit globular cytosolic structures, which are usually indicative of octameric ring formation (Bornholdt et al., 2013).

### VP40 CAN DEEPLY PENETRATE THE PLASMA MEMBRANE AND INDUCE MEMBRANE CURVATURE CHANGES

To dig deeper into the mechanism of VP40-mediated membrane penetration, a Trp residue was introduced at Ala^299^ within the hydrophobic loop (Soni et al., 2013). Trp is advantageous as its ability to fluoresce can be used in combination with brominated lipids to determine the depth of penetration of peripheral proteins. Brominated lipids are available with bromine on different positions of the acyl chain so their ability to quench Trp in the lipid-binding site can be compared using depth-dependent fluorescence quenching profiles (DFQP). Since the depth of bromine positions have been calibrated using X-ray diffraction (McIntosh and Holloway, 1987; Kleinschmidt and Tamm, 1999), the depth of Trp was determined using the distribution analysis (DA) method (Ladokhin, 1997, 1999). It is important to note that VP40 and A299W had similar affinity for PS liposomes, PM localization, oligomerization, and A299W retained the ability to form VLPs. Lipids with bromine on the 9th and 10th carbons were most efficient at quenching A299W indicating their proximity to the residues in this hydrophobic loop region. The DA calculation determined the mean depth of VP40 penetration to be 8.1 Å, which is more than halfway through one leaflet of the bilayer. *In vitro* and cell experiments demonstrated that introducing less hydrophobic mutations into the A299W construct (I293A/A299W and L295A/A299W) lead to poor penetration into the hydrocarbon core, reduced PM localization, and very low levels of VLP formation (Soni et al., 2013). These data indicated that loss of perhaps even one key hydrophobic contact would reduce viral egress. Electron microscopy (EM) and giant unilamellar vesicle (GUV) imaging demonstrated that VP40 induced negative membrane curvature changes in a PS-dependent manner. This type of curvature generation is consistent with VP40 pushing the inner leaflet of the PM out of the cell to form a VLP. As expected from the other assays, hydrophobic mutations reduced the ability of VP40 to induce membrane curvature changes. These studies suggested VP40 plays a major role in remodeling the PM shape to form the filamentous VLP.

### VP40 MEMBRANE INTERACTION SUMMARY

These cellular and *in vitro* membrane-binding studies on VP40 have revealed some of the important processes that regulate VP40 PM localization and VLP formation. VP40 has both electrostatic and hydrophobic components to its membrane interaction that are important to VLP formation. Notably, the C-terminal domain of VP40 deeply penetrates the PM, which can induce negative curvature changes in synthetic membranes (Soni et al., 2013). Membrane association of VP40 also appears to be a prerequisite to inducing hexamerization (Scianimanico et al., 2000; Adu-Gyamfi et al., 2012a, 2013; Bornholdt et al., 2013). On the other hand, taken together these studies demonstrate there is still dearth of information on how VP40 associates with the PM inner leaflet. Remarkably, it appears VP40 lipid selectivity has not been systematically and carefully investigated. This is of utmost importance when the complex chemical composition of the PM inner leaflet is considered. Moreover, many peripheral proteins have been shown to undergo a complex set of interactions with membrane bilayers in order to achieve membrane docking and membrane curvature generation (Balla, 2005; Lemmon, 2008; Moravcevic et al., 2012; Scott et al., 2012; Mim and Unger, 2012; Stahelin et al., 2014). Thus, more detailed biochemical and biophysical investigation at the membrane interface is warranted to elucidate how VP40–lipid interactions mediate filamentous particle formation.

## VP40 STRUCTURES REVEAL THE BASIS OF OLIGOMERIZATION AND MEMBRANE BINDING

**FIGURE 2:**
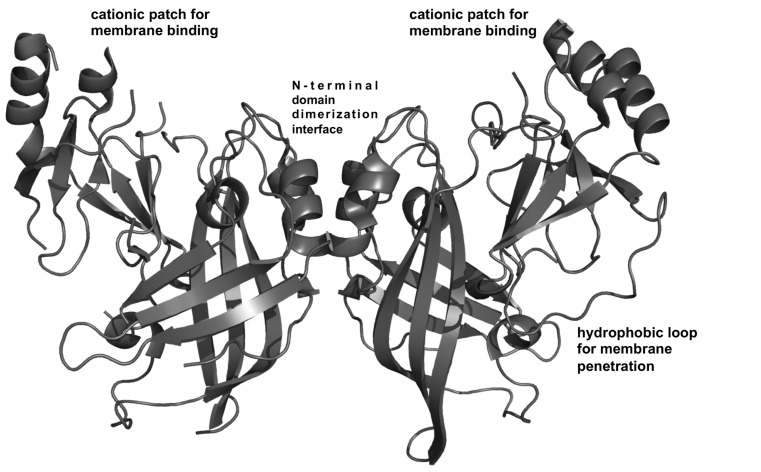
**VP40 structural analysis**. VP40 is a dimer (Bornholdt et al., 2013) that is the building block for VP40 filament formation. Dimerization is mediated by a hydrophobic N-terminal domain interface (Bornholdt et al., 2013). The dimeric structure revealed a robust cationic patch exposed on one face of the dimer (Bornholdt et al., 2013) Mutation of Lys residues in this C-terminal domain cationic patch greatly reduced PM localization and budding in cells and is surmised to mediate membrane binding (Bornholdt et al., 2013). Adjacent to the cationic patch is a hydrophobic loop in the C-terminal domain that mediates membrane insertion of VP40, an important step for VP40 PM binding and membrane curvature generation (Adu-Gyamfi et al., 2013; Soni et al., 2013).

Recently, Bornholdt et al. (2013) solved several EBOV VP40 high-resolution structures that demonstrated VP40 can transform into multiple arrangements. These distinct VP40 structures have separate but critical roles in the life cycle of the virus (Bornholdt et al., 2013). VP40 was revealed to be a dimer (see **Figure 2**) where each dimer serves as a building block that can further assemble into a flexible filamentous matrix protein structure (Bornholdt et al., 2013). VP40 dimerization is mediated by a hydrophobic N-terminal domain (NTD) interface, which when mutated, leads to abolishment of PM localization and VLP formation (Bornholdt et al., 2013). VP40 dimers, which are oblong, form linear filaments that have a hydrophobic C-terminal domain (CTD) interface (Bornholdt et al., 2013). These hexamers may further multimerize into a lattice or filamentous structure (Bornholdt et al., 2013) that regulates EBOV budding and virion shape. A third interface containing Trp^95^ was revealed following incubation with dextran sulfate, which was used as an anionic membrane mimetic (Bornholdt et al., 2013). This third interface is also important to assembly and budding as previous mutation of Trp^95^ diminished oligomerization and egress (Hoenen et al., 2010). VP40 dimers and hexamers may interact laterally through this third interface (Trp^95^), which would be more accessible after membrane binding, to form a highly dense filament (Bornholdt et al., 2013). VP40 can also form an octameric ring, which binds RNA and functions in viral transcription inside the cell (Bornholdt et al., 2013). The octameric ring is not recruited into VLPs or infectious virions and if or how it interacts with cellular membranes is still unknown.

### VP40 DIMERS INTERACT THROUGH A HYDROPHOBIC NTD INTERFACE

The interface in the NTD of VP40 buries ~1500–1700 Å^2^ in the dimer (Bornholdt et al., 2013). This involves α-helices containing residues 52–65 and 108–117 (Bornholdt et al., 2013). The NTD interactions are primarily hydrophobic and have few H-bonds. This interaction network specifically involves Ala^55^, His^61^, Phe^108^, Thr^112^, Ala^113^, Met^116^, and Leu^117^ (Bornholdt et al., 2013). Leu^117^ was found to be a key residue in the NTD interaction. This Leu extends into a hydrophobic pocket (Ala^55^, His^61^, Met^116^, and Phe^108^) to lock the dimer together (Bornholdt et al., 2013). This NTD interface is also much different than the interface that mediates the RNA-binding octameric ring (Gomis-Rüth et al., 2003). In order to form the octameric ring from the VP40 dimer, large conformational changes are necessary including movement of the C-terminal domain as well as extensive unraveling of the N-terminal 69-amino acids (Bornholdt et al., 2013). T112R and L117R abrogate the dimer formation and form monomers and octameric ring structures (Bornholdt et al., 2013). Neither mutant is released as VLPs and PM localization for the most part is undetectable (Bornholdt et al., 2013).

### VP40 DIMERS FORM FILAMENTOUS HEXAMERS THROUGH A HYDROPHOBIC CTD INTERFACE

In all crystal structures of VP40 solved with the CTD visible, the CTD formed CTD-to-CTD interfaces that are hydrophobic and involved residues Leu^203^, Ile^237^, Met^241^, Met^305^, and Ile^307^ (Bornholdt et al., 2013). These interactions allowed VP40 dimers to form filamentous structures. The structure revealed this interface to have torsional motion, perhaps an important property in forming a flexible and filamentous structure from PM lipids (Bornholdt et al., 2013). Met^241^ was mutated as it resides at the center of the interface but is not essential to the fold of the CTD. M241R was generated, is dimeric in solution, and does not bud VLPs (Bornholdt et al., 2013). However, membrane ruffling was observed for M241R indicating the dimer is the building block to induce membrane curvature changes (Bornholdt et al., 2013). The M241R structure was solved to 4.15 Å resolution, which further demonstrated that this mutation did not disrupt the fold of the dimer but forced packing of dimers with a twisted interface relative to that of WT (Bornholdt et al., 2013). In the twisted M241R structure, the CTD interface harbored residues Leu^203^ and Ile^307^ at the center of the interface rather than Met^241^ (Bornholdt et al., 2013). M241R also prevented rotation about the CTD–CTD interface (Bornholdt et al., 2013) suggesting that a fairly precise hexamer with proper torsional motion is necessary to form filamentous particles via PM bending.

### VP40 OCTAMERIC RINGS CAN BE INDUCED BY THE I307R MUTATION

I307R was made to investigate the CTD interface further but demonstrated extensive formation of octameric rings that are bound to nucleic acid (Bornholdt et al., 2013). In cells, I307R is not detectable at the PM but instead forms globular structures that are perinuclear (Bornholdt et al., 2013). Octameric ring formation thus blocked budding of VLPs (Bornholdt et al., 2013). Gln^309^ was mutated as a control for Ile^307^ and behaved similar to WT (Bornholdt et al., 2013) while R134A was prepared to knockout RNA binding (Gomis-Rüth et al., 2003). R134A behaved similar to WT in PM localization and budding and formed a dimer in solution (Bornholdt et al., 2013). The double mutant, R134A/I307R, was made to prevent CTD-mediated filament assembly where the R134A component would prevent RNA binding and thus octameric ring formation. As expected, R134A/I307R is a nucleic-acid-free dimer in solution and no longer forms globular structures that are perinuclear in cells (Bornholdt et al., 2013). Instead, this double mutant translocates to the PM but does not bud or form VLPs as it cannot form effective hexamers (Bornholdt et al., 2013). While PM localization is evident for R134A/I307R, membrane ruffling is not like that of the M241R mutation (Bornholdt et al., 2013). Overall, their modeling studies suggested that I307R prohibits CTD-to-CTD interactions while M241R permits them but with twisted and defective interactions (Bornholdt et al., 2013).

### A CATIONIC C-TERMINAL DOMAIN INTERFACE IS REVEALED

These new VP40 structures also revealed a disordered loop in the C-terminal domain that contains several positively charged residues (Bornholdt et al., 2013). A cationic patch in the CTD is electrostatically important for binding the PM and forming VLPs (Bornholdt et al., 2013). The basic patch in the CTD is exposed and made up of six Lys residues (Lys^221^, Lys^224^, Lys^225^, Lys^270^, Lys^274^, and Lys^275^; Bornholdt et al., 2013) some of which are in a loop region not previously observed in the original structure (Dessen et al., 2000). This patch is also conserved across the five species of EBOV. A 10-amino-acid loop deletion (Δ^221-229^) that included removal of Lys^221^, Lys^224^, and Lys^225^ lead to abrogation of budding but the dimeric structure was still maintained (Bornholdt et al., 2013). Mutations of Lys^224^ and Lys^225^ to Met or Glu abrogated PM localization and budding but when mutated to Arg retained WT function (Bornholdt et al., 2013). K274E/K275E exhibited little formation of VLPs but some translocation to the PM interface (Bornholdt et al., 2013). Restoration of positive charge to this region (K274R/K275R) was able to recover VLP formation at WT levels (Bornholdt et al., 2013). These C-terminal domain basic charges are necessary for budding and it is important to note that these cationic residues are not essential to the structure or any known protein–protein interfaces. It should also be noted that these loop regions harbor several Asn and Ser residues that could H-bond with PM lipid headgroups. The cationic residues are exposed on one side of the dimer and also adjacent to the hydrophobic residues previously shown to penetrate into the PM hydrocarbon core (**Figure 2**). Together they may form a robust interface for PM docking (Stahelin, 2014).

## CONCLUSION AND FUTURE PERSPECTIVES

### VP40 MEMBRANE BINDING MODEL

**FIGURE 3:**
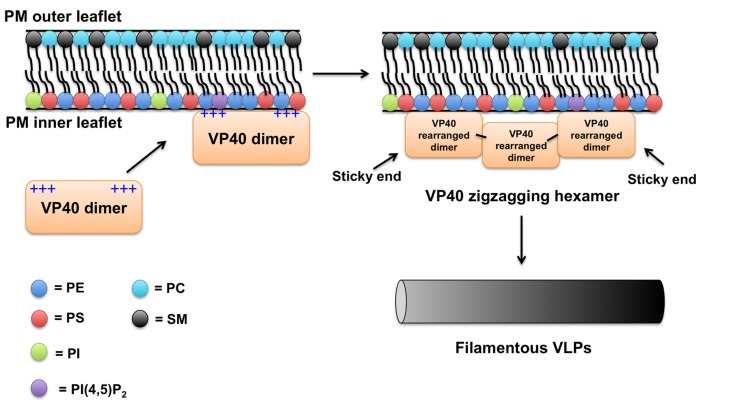
**Plasma membrane binding model for VP40**. Considering previous studies and available data on VP40 structure and membrane-binding properties a simplified model of VP40 association with the PM is proposed. The VP40 dimer, which contains a large cationic solvent exposed patch in the C-terminal domain likely mediates the association of VP40 with the highly anionic interface of the PM. VP40 interactions with PS (Ruigrok et al., 2000; Scianimanico et al., 2000; Adu-Gyamfi et al., 2013; Soni et al., 2013) and perhaps other lipids such as PIPs induce a conformational change of VP40 rearranging the dimers into a zigzagging hexamer (Bornholdt et al., 2013). The VP40 dimers can interconnect through the CTD to form linear hexamers as well (Bornholdt et al., 2013). These hexamers have “sticky” ends that can further interact to form long VP40 filaments (Bornholdt et al., 2013). Lateral interactions between VP40 filaments are also possible and can occur between dimers and hexamers (Bornholdt et al., 2013). Note, in this model a VP40 monomer would not robustly interact with the PM (Bornholdt et al., 2013) presumably due to weak affinity or lack of transport to the PM. Hydrophobic interactions between the more liberated (Bornholdt et al., 2013) C-terminal domain hydrophobic loop (Jasenosky et al., 2001; Adu-Gyamfi et al., 2013; Soni et al., 2013) and the PM hydrophobic core act to increase the membrane residence time of VP40. As VP40 assembles on the inner leaflet of the PM, the C-terminal hydrophobic residues are able to penetrate ~8.1Å. VP40 oligomers are able to induce budding (negative membrane curvature generation; Soni et al., 2013) of a filamentous particle that eventually will undergo scission and be released.

VP40 may be somewhat similar to the VSV matrix protein, which has been shown to engage in both electrostatic and hydrophobic interactions with lipid membranes (Ye et al., 1994; Gaudier et al., 2002). Taking into account the lipid-binding studies performed on VP40 and the new structural information that has become available, a rough membrane-binding model can be proposed (see **Figure 3**). In the first step of PM binding, the electrostatic patch in the C-terminal domain likely mediates the membrane association step with the highly anionic PM interface. The VP40 dimer would be advantageous in this regard as it would have enhanced avidity for the membrane compared to a VP40 monomer with only one cationic patch. Whether or not this patch directly coordinates specific lipid headgroups is unknown, but in the case of PS binding would induce VP40 hexamerization (Scianimanico et al., 2000). This structural rearrangement would allow the penetration and docking of the C-terminal hydrophobic loop into the PM, so VP40 could lock in the membrane for the ride out of the cell.

How does PM binding induce VP40 hydrophobic penetration? This is still unknown but some peripheral proteins have been shown to coordinate a lipid headgroup to decrease the desolvation penalty associated with hydrophobic membrane insertion (Stahelin et al., 2002, 2003a). The VP40 hexamer or longer filament that concatenates may then provide the energetic force necessary to induce negative membrane curvature generation from the PM interface. The studies by Bornholdt et al. (2013) provided a very attractive model for understanding this process as the M241R mutation that alters torsional motion of the VP40 hexamer demonstrates extensive membrane protrusions from the PM. However, this mutant lacked effective VLP formation suggesting an inability to undergo scission (Bornholdt et al., 2013). Thus, the VP40 hexamer may be the driving force for negative membrane curvature generation and formation of PM protrusions but without proper torsional motion across the CTD interface VP40 may not effectively further multimerize longitudinally or laterally to either drive effective VLP formation or alter interactions with the ESCRT complex, which may be needed for efficient VLP release. The elegant proposal by Bornholdt et al. (2013) which accounted for the precise location of VP40 between the viral lipid envelope and nucleocapsid, proposed that VP40 C-termini point out from both planes of a VP40 lattice engaging both the lipid envelope and viral RNA. This model may also help explain how VP40 achieves initial membrane curvature generation as some C-termini would be deeply engaged in the membrane hydrophobic core while the C-termini in the opposite plane would interact with RNA (Bornholdt et al., 2013). As VP40 interacts both laterally and longitudinally beneath the PM (Bornholdt et al., 2013), the dual engagement of membrane and RNA by two C-terminal interfaces may provide the driving force for PM protrusion and virus structure.

Protein–protein interactions may also play an important role in VP40 assembly and egress and cannot be discounted in these models. VP40 may associate with actin (Han and Harty, 2005), IQGAP1 (Lu et al., 2013), Sec24C (Yamayoshi et al., 2008), and microtubules (Ruthel et al., 2005). Additionally, actin has been shown to regulate ballistic motion of VP40, where inhibition of actin polymerization lead to constrained diffusion of VP40 molecules on the PM (Adu-Gyamfi et al., 2012b). The roles these interactions play are only beginning to be unraveled and a comprehensive view of VP40–protein and VP40–lipid interactions could help resolve the molecular details of EBOV assembly and budding.

### IS VP40 AN ANIONIC CHARGE SENSOR OR DOES IT SPECIFICALLY BIND LIPID HEADGROUPS?

To date, the lipid selectivity and membrane affinity of VP40 is still unknown. Several studies, as outlined in this review, have shown that VP40 can associate with, penetrate, and hexamerize in response to interactions with PS-containing liposomes. However, many peripheral proteins have distinct lipid selectivity and binding sites for lipid headgroups including those of PS and PIPs (Cho and Stahelin, 2005; Lemmon, 2008; Huang et al., 2011; Lucas and Cho, 2011). Additionally, many lipid-binding proteins are coincidence detectors and interact with two distinct lipid headgroups or a lipid and membrane physical property such as charge or curvature (Balla, 2005; Lemmon, 2008; Mim and Unger, 2012; Moravcevic et al., 2012; Scott et al., 2012; Stahelin et al., 2014). More systematic analysis of VP40 lipid selectivity is needed to discover the optimal lipid composition that VP40 interacts with. This is especially important considering the complex chemistry of the PM lipid environment including important PM lipids, such as PS, PE, PI, cholesterol, and PIPs. Additionally, lipid acyl chain length and saturation can vary at the PM, which may also play an important role in VP40 PM detection and membrane curvature generation. As a VP40 budding particle is formed relatively flat membrane curvature is generated along the particle trajectory, while the neck region extending from the PM contains a region of negative curvature. Perhaps, the VP40 hexamers and filaments that form becomes activated in the sense they assemble on the more negatively curved membrane neck region. In other words, perhaps distinct VP40 structures have selectivity for the shape of the PM. In time these questions will likely be answered but will require robust biochemical and biophysical investigation of VP40 lipid-binding and -bending properties. These analyses should also include the lipid-binding determinants of VP40 zigzagging hexamer formation.

For the sake of comparison, it should be noted that the matrix domain of HIV-1, which has been known to associate with PM PI(4,5)P_2_ (Ono et al., 2004) has more recently been shown to undergo a complex set of interactions with the PM that includes roles for lipid acyl chain saturation (Dick et al., 2012), lipid headgroup structure (Vlach and Saad, 2013), and protein oligomerization (Dick et al., 2013). Notably, PS/PE/PC have been shown to bind the HIV-1 matrix domain at a different site than that of PI(4,5)P_2_ (Vlach and Saad, 2013). HIV-1 matrix has also been shown to be sensitive to the saturation on the acyl chains of PS and cholesterol content in lipid vesicles (Dick et al., 2012). Thus, viral matrix proteins likely use a complex set of chemical interactions with the membrane to regulate the spatial and temporal assembly of viral particles. This may also be important when considering formation of long and flexible EBOV filamentous particles.

### CAN VP40 INDUCE FLIPPING OF PS?

A number of viruses including EBOV have now been shown to utilize PS exposed on the outer membrane envelope of the virus to facilitate entry into target cells (Soares et al., 2008; Jemielity et al., 2013; Morizono and Chen, 2014). PS interacts with the TIM-1 receptor on human cells and inhibition of PS exposure or PS availability to interact with the TIM-1 receptor, may be of great therapeutic value in a number of viral infections (Jemielity et al., 2013; Moller-Tank et al., 2013; Morizono and Chen, 2014). Strikingly, VP40 VLPs lacking the GP are able to enter target cells in a PS-dependent manner (Jemielity et al., 2013; Moller-Tank et al., 2013). This suggests that the information encoding PS exposure in VLPs may be encoded in VP40 alone. The PM asymmetry in eukaryotic cells is kept intact by ATP-dependent aminophospholipid flippases (Daleke, 2007; Baldridge and Graham, 2012), which keep PE and PS concentrated on the PM inner leaflet. Studies on erythrocytes have shown that >96% of PS is on the inner leaflet (Zachowski, 1993) meaning only 4% or less is exposed to the outer leaflet. While the percentage of PS exposure natively on most cell types is not known, it is not expected to be high. Is 4% PS on the outer filovirus envelope enough molecules of PS to facilitate entry? Perhaps, however, how, when, and how much PS is exposed on viral particles is unknown. This leads the author to speculate that PS may become more significantly exposed on VP40 VLPs or EBOV virions. Several possibilities exist: (i) Curvature generation in vesicular transport from the Golgi has been shown to expose (flip) PS in budding vesicles (Xu et al., 2013), which may also occur as the VP40 bud site is formed. (ii) VP40 VLPs may restrict or limit incorporation of flippases and along with lack of ATP in the VLPs leads to some loss of asymmetry. (iii) Calcium-dependent scramblases may become active in the plasma membrane (Zhou et al., 1997) or in VLPs. Lipid scramblases are still a controversial subject but in some studies have been shown to randomize the lipid distribution across the PM. Given the important nature of PS in viral entry, these are some potential lines of investigation to explore. We still have a lot to learn regarding VP40-mediated assembly of the EBOV envelope but there are many exciting endeavors to pursue in the lipid field along the way.

## Conflict of Interest Statement

The author declares that the research was conducted in the absence of any commercial or financial relationships that could be construed as a potential conflict of interest.
